# High accuracy of pooled DNA genotyping by 2b-RAD sequencing in the Pacific white shrimp, *Litopenaeus vannamei*

**DOI:** 10.1371/journal.pone.0236343

**Published:** 2020-07-30

**Authors:** Juan Sui, Sheng Luan, Ping Dai, Qiang Fu, Xianhong Meng, Kun Luo, Baoxiang Cao, Jie Kong

**Affiliations:** 1 Key Laboratory for Sustainable Utilization of Marine Fisheries Resources, Ministry of Agriculture, Yellow Sea Fisheries Research Institute, Chinese Academy of Fishery Sciences, Qingdao, China; 2 Laboratory for Marine Fisheries Science and Food Production Processes, Qingdao National Laboratory for Marine Science and Technology, Qingdao, China; National Cheng Kung University, TAIWAN

## Abstract

Using pooled DNA genotyping to estimate the proportional contributions from multiple families in a pooled sample is of particular interest for selective breeding in aquaculture. We compared different pooled libraries with separate 2b-RAD sequencing of *Litopenaeus vannamei* individuals to assess the effect of different population structures (different numbers of individuals and families) on pooled DNA sequencing, the accuracy of parent sequencing of the DNA pools and the effect of SNP numbers on pooled DNA sequencing. We demonstrated that small pooled DNA genotyping of up to 53 individuals by 2b-RAD sequencing could provide a highly accurate assessment of population allele frequencies. The accuracy increased as the number of individuals and families increased. The allele frequencies of the parents from each pool were highly correlated with those of the pools or the corresponding individuals in the pool. We chose 500–28,000 SNPs to test the effect of SNP number on the accuracy of pooled sequencing, and no linear relationship was found between them. When the SNP number was fixed, increasing the number of individuals in the mixed pool resulted in higher accuracy of each pooled genotyping. Our data confirmed that pooled DNA genotyping by 2b-RAD sequencing could achieve higher accuracy than that of individual-based genotyping. The results will provide important information for shrimp breeding programs.

## Introduction

The conventional selection breeding system of aquatic animals is family based, in that it utilizes multitrait selection based on communal rearing of physically tagged families and pedigree records [1–3]. Thousands of individuals per generation would be tagged to carry out multitrait and multienvironment testing at the same time. However, the process is costly and laborious. More importantly, families need to be cultured separately until individual tagging, which not only occupies a large number of farming facilities and affects the growth rate of shrimp but also leads to common environmental effects in genetic evaluation and affects the accuracy of breeding value estimation [4, 5]. Individual genetic markers are an effective tagging method which could break the limitation of environmental conditions. SNPs (single nucleotide polymorphisms) have been widely used because of their abundance, ease and high throughput of scoring [6]. However, there are thousands of selection candidates and test individuals in aquaculture breeding, which make genotyping costly even though the cost of individual-based genotyping has dramatically decreased.

Selective breeding of penaeid shrimp over the last 20 years has guaranteed a sustainable shrimp aquaculture industry globally, which was worth over US 800 million dollars in 2017 [3, 7, 8]. The Pacific white shrimp *Litopenaeus vannamei* is the most important cultured shrimp worldwide, accounting for ~80% of total cultured penaeid shrimp production. Of all the aquaculture genomes, the shrimp genome is perhaps the hardest to deal with because of the difficulty in isolating high molecular weight DNA due to enhanced DNase activity, the large chromosome number, and high levels of heterozygosity and repetitive elements [9]. The *L*. *vannamei* genome size was measured to be 2.45 Gb by flow cytometry [10]. The complexity and large size of the *L*. *vannamei* genome increase the difficulty of DNA sequencing. Restriction-site associated DNA (RAD) sequencing can effectively reduce the complexity of the genome [11–13]. It has become an economical and efficient method for SNP discovery and genotyping [14, 15] Recently, an improved RAD-seq technology, called 2b-RAD sequencing, was developed with high accuracy in genotyping [16]. Library construction for 2b-RAD sequencing is simple and fast, and its most prominent feature is that the tag density could be adjusted by adding selective bases to the joints, which means that the cost could be easy to control [16]. It has been widely used in SNP genotyping, genetic map construction and genomic selection for aquatic animals [17–20].

Kinghorn et al. [21] reported a method by which the proportional contributions and trait information of families contributing to the mixed populations could be inferred from the changes in population allele frequency of SNP markers. The correlation between allele frequency estimates derived from individual genotyping and pooled DNA genotyping could reach 99% based on mathematical derivations. This method provides a useful tool for communal rearing at an early age in aquatic breeding systems. However, the most important concern in practical application is the accuracy of pooled DNA sequencing. Several recent studies have illustrated the potential of pool- versus individual-based experimental designs for identifying and quantifying SNP variants. These studies have demonstrated that pooled DNA sequencing could generate satisfactory accuracy and repeatability at a quite lower cost than that of individual sequencing in *Arabidopsis halleri* (Brassicaceae) [22], pine processionary moth (*Thaumetopoea pityocampa*) [23], *Drosophila melanogaster* [24, 25], Atlantic salmon (*Salmo salar* L.) [26] and mathematical deduction [23, 27].

Barratt et al. [28] found that small pools with approximately 50 individuals would get more information than a few large pools. Thus, in this study, we applied 2b-RAD sequencing to compare four different small pooled libraries of up to 53 individuals with separate sequencing of *L*. *vannamei* individuals to assess 1) the effect of population structure (different numbers of individuals and families) on pooled DNA sequencing; 2) the accuracy of parent sequencing of the DNA pools and 3) the effect of SNP numbers on pooled DNA sequencing. The results showed that pooled 2b-RAD sequencing provides a highly accurate assessment of population allele frequencies.

## Materials and methods

### Animals

Animals used in this study were from the fourth generation of a breeding population of *L*. *vannamei* that was cultured in Xinhai Aquatic Biological Technology Co., Ltd. (Hebei province, China). Fifty-three individuals were randomly selected from ten families for DNA pool construction. Two of the 10 families were paternal half-sib families, and there were a total of 19 parents of the 10 families, including 9 sires and 10 dams. Muscle tissues of the 72 samples (53 individuals and 19 parents) were dissected and frozen in a -80°C freezer.

### DNA isolation and DNA pooling strategy

Total genomic DNA was extracted using a genomic DNA extraction kit for marine animal tissues (Tiangen). DNA quality was detected by 1% agarose gels. DNA concentration of each individual was accurately quantified by Qubit 3.0 Fluorometer (Invitrogen) and normalized to 50 ng/ml.

The number of individuals and families included in each pool is shown in Table 1. A total of four DNA pools were constructed using 53 progeny individuals from 10 families. Pool 1 consisted of three families (F1-F3), each with five individuals. Pool 2 consisted of three families (F1-F3), each with ten individuals, the 30 individuals in pool 2 contained the 15 individuals in pool 1. Pool 3 consisted of six families (F1-F6), each with five individuals, the 30 individuals in pool 3 were consistent with eight individuals in pool 1 and pool 2, respectively. Pool 4 consisted of ten families and 53 individuals in total, of which 3 families had 10 individuals in each family (F1-F3), 3 families had 5 individuals in each family (F4-F6), 2 families had 3 individuals in each family (F7-F8), and 2 families had 1 individual in each family (F9-F10). Pool 4 included all the individuals used in Pool 1, Pool 2 and Pool 3. The difference between pool 1 and pool 2 was that the family number in each pool was the same, but the individual number was different. The difference between pool 2 and pool 3 was that the individual number in each pool was the same, but the family number was different. Pool 4 represented a random sampling from a large number of samples.

**Table 1:** Number of individuals and families included in each pool.

Poo1 Family	F1	F2	F3	F4	F5	F6	F7	F8	F9	F10	Total
Pool 1	5	5	5								15
Pool 2	10	10	10								30
Pool 3	5	5	5	5	5	5					30
Pool 4	10	10	10	5	5	5	3	3	1	1	53

### DNA isolation, library construction and sequencing

Each individual provided 6 ng DNA to the pool. Each pool was evenly mixed and stored at -20°Cfor 2b-RAD library construction. Fifty-three individuals used in the four pools were also genotyped to obtain the ‘true’ allele frequency for each locus in a population. The libraries of 53 progeny samples, 19 parent samples and 4 pooled samples were prepared for 2b-RAD sequencing by Qingdao OE Biotech Co., Ltd. (Qingdao, China), according to the method described by Wang et al. [16].

The quality of all amplicon libraries was checked on a 1.8% agarose gel and then purified using the SPRI select purification kit (Beckman Coulter, Pasadena, CA). The concentration of the purified libraries was quantified using a Qubit dsDNA BR Assay Kit (Invitrogen, USA) and Mx3000P qPCR Instrument, and the quality was checked on an Agilent 2100 Bioanalyzer (Agilent Technologies, Santa Clara, California, USA). Finally, all libraries were sequenced on an Illumina NextSeq500 platform (Illumina, San Diego, CA) using 50 bp single-end sequencing (v2 chemistry, high output kit—50 cycles). Each individual was sequenced to a depth of 15 × and each pool was sequenced to a depth of 100 ×。

### Genotyping and quality control

Raw reads were processed using a custom Perl script to trim adaptor sequences. The terminal 3-bp positions were also excluded from each read to eliminate artifacts that might have arisen from ligation sites, and the final read length obtained was 33-bp. Reads with ambiguous bases (N) > 8%, poor quality (15% nucleotide positions with a Phred quality score < 30), or without restriction sites were removed.

The BsaXI tags in the genome of *L*. *vannamei* (GenBank accession numbers QCYY01000001-QCYY01004682) [10] were extracted based on the enzyme’s recognition site, which served as a reference for SNP discovery. High-quality reads of each sample were aligned to the genome reference using SOAP2 [29] with the following parameters: r = 0, M = 4, v = 2. The aligned data for each sample were then used for SNP detection by the RAD typing [30] program with default parameters.

To obtain robust results in the subsequent analyses, the following stringent criteria were applied for SNP filtering: (1) Loci shared by < 80% were eliminated. (2) SNPs with a minor allele frequency (MAF) < 0.01 were discarded. (3) Polymorphic loci with more than two alleles possibly derived from sequencing or clustering errors were excluded. (4) Tags with more than two SNPs were excluded. A SNP that passed the above-mentioned criteria was considered a putative SNP for further analyses.

### Data analysis

The consistency of allele frequencies between individual- and pooled- sequencing was investigated, as reflected by the Pearson’s correlation coefficient of allele frequencies at all loci. The impact of different pooling strategies including different numbers of individuals and families mixed in the pool was investigated. The consistency of allele frequencies between parents and offspring individuals or offspring pools were investigated. The correlation between allele frequencies of the four different pools or individuals in the pools and their corresponding parents were analyzed. The allele frequencies of the parents of each pool were calculated by their individual genotyping results. The genetic relationship of 53 progeny and 19 parents was first identified by colony software (version 2.0.3.4) according to the results of SNP genotyping. An individual belonging to pool 4 was inconsistent with the record's dam, so among the parents of pool 4, the dam was removed.

To analyze the variation in allele frequency of the breeding population, only some individuals can be selected for mixed-pool sequencing. Whether different numbers of individuals can effectively represent the real allele frequency is worth studying. Thus, the correlation between the allele frequency obtained in different pools and all individuals was analyzed. Relative error was computed according to the formula Error = ((freq_pool_-freq_indi_)/freq_indi_)^2 [24].

In general, with more families mixed in a pool, more markers are needed to distinguish the family contribution [21]. The effect of SNP number on the consistency of allele frequencies between pools and the constituent individuals was investigated in this study. We selected 500–28,000 SNPs to test the effect of the SNP number on the accuracy of pool sequencing using the following procedure. First, all 28,882 SNPs were arranged in a fixed order. Second, we selected at least 500 SNPs, and each selection increased by 500, up to 28,800. After the SNP number was determined, for example, 500, it was regarded as a “window”. The “window” started from the first SNP, and then formed a “step” of all SNPs in turn. The length of each step was 100 SNPs. Third, the correlation coefficients of allele frequencies between pool- and individual- sequencing in each “window” and “step” were calculated. After completion of the “window” and “step” calculations, the mean values of all correlation coefficients obtained in the “window” after each “step” were calculated as the correlation coefficients between pool- and individual- sequencing under the given SNP number.

### Verification of pool sequencing repeatability

In the process of pool construction and sequencing, compounded error might be introduced into final allele frequency estimates from unavoidable technical and biological errors, including variability in rearing, pooling, DNA extraction, PCR reactions and sequencing. Although the stability of RAD library construction and sequencing process has been tested in many aspects, such as genotyping [31] and tags [13], it is still necessary to verify the repeatability of pool sequencing method in this study because of the uncertainties mentioned above. Considering the purpose and cost, we performed two verifications as follows: 1) pool 3 was repeated three times from DNA extraction to genotyping to check the consistency of the results; 2) another 30 individuals from 10 families were reselected from 53 individuals to form pool 5 for three repeats (14 individuals were consistent with pool 2 and 15 individuals were consistent with pool 3) to check the consistency of pool with the same number of individuals but different sources. The process of DNA isolation, library construction, sequencing, genotyping, quality control and data analysis was exactly as mentioned before.

## Results

### Genotyping RAD alleles

Sequencing of 76 libraries yielded a total of 2.66 billion raw reads, distributed as 0.27 and 2.39 billion across parents and offspring respectively. Quality filtering of the raw reads reduced the number of reads by 31.71% (loss of 0.84 billion reads). The average number of unique tags was 286,602 after removing the unique tags whose sequencing depth was less than 3. After filtering, there were 28,882 quality SNPs in all samples including pools and individuals.

### Effect of population structure

The consistency and relative error of allele frequencies between different pools and all individuals (53) and different pools and the constituent individuals are shown in Table 2. For pool 1 of 15 individuals from 3 families, the correlation coefficients of allele frequencies of different pools with all individuals were 0.9831–0.9836 and were 0.9863–0.9876 with the constituent individuals; for Pool 2 of 30 individuals from the same 3 families, the correlation coefficients of allele frequencies with all individuals were 0.9869–0.9874 and were 0.9902–0.9909 with the constituent individuals; for Pool 3 of 30 individuals from 6 families, the correlation coefficients of allele frequencies with all individuals were 0.9918–0.9922 and were 0.9933–0.9936 with the constituent individuals; for Pool 4 of 53 individuals from 10 families, the correlation coefficients of allele frequencies with all individuals (the constituent individuals) were 0.9934–0.9936. With the increase of families and individuals, the allele frequency correlation coefficient between pool and individual sequencing increased. The correlations between allele frequencies of each pool and its constituent individuals were slightly higher than those of each pool and 53 individuals (pool 4 excluded). When the family number was fixed (pool 1 and pool 2), the Pearson’s coefficient of allele frequencies between pool and individual sequencing was higher when the individual number was higher. When the individual number was fixed (pool 2 and pool 3), the Pearson’s coefficient of allele frequencies between pool and individual sequencing was higher when the family number increased. The Pearson’s coefficients increased as the number of individuals and families increased (S1 Table and S2 Table).

**Table 2:** Comparison of allele frequency estimates between pool DNA and individuals.

Pool	Allele	4 pools and 53 individuals		4 pools and the constituent individuals	
		Pearson’s coefficient	Relative error	Pearson’s coefficient	Relative error
1	A	0.9836	1.5099±13.0022	0.9876	0.3718±2.2273
	T	0.9831	1.3733±11.2925	0.9872	0.4451±3.2501
	C	0.9832	0.8099±6.7785	0.9863	0.2290±1.6713
	G	0.9832	0.8660±9.5250	0.9870	0.2271±1.9190
2	A	0.9873	1.2037±8.2768	0.9908	0.4570±3.0972
	T	0.9870	1.0190±6.4142	0.9909	0.4176±2.6806
	C	0.9869	0.7160±5.9080	0.9902	0.2817±2.0893
	G	0.9874	0.6616±6.0681	0.9909	0.2562±2.3916
3	A	0.9922	0.9675±8.1875	0.9936	0.4907±2.8691
	T	0.9921	0.8381±5.7278	0.9936	0.4510±2.4208
	C	0.9922	0.6149±5.0877	0.9934	0.3095±2.0098
	G	0.9918	0.6426±5.3051	0.9933	0.3346±2.2224
4	A	0.9936	0.6970±4.9239	0.9936	0.6970±4.9239
	T	0.9934	0.6876±4.6355	0.9934	0.6876±4.6355
	C	0.9935	0.4877±4.3982	0.9935	0.4877±4.3982
	G	0.9935	0.4538±4.2453	0.9935	0.4538±4.2453

For the comparison of the 4 different pools and all individuals, the relative error was 0.8099–1.5099 for pool 1, 0.6616–1.2037 for pool 2, 0.6149–0.9675 for pool 3 and 0.4538–0.6970 for pool 4. The relative error and its standard error decreased as the number of individuals and families increased (Table 2). For the comparison of the 4 different pools and the constituent individuals, the relative error was obviously lower than that of the former, which was 0.2271–0.4451 for pool 1, 0.2562–0.4570 for pool 2, 0.3095–0.4907 for pool 3, and 0.4538–0.6970 for pool 4. When the family number was fixed (pool 1 and pool 2), the relative error was higher when the individual number increased. When the individual number was fixed (pool 2 and pool 3), the relative error was higher when the family number increased (S1 Table and S2 Table).

### Estimation of allele frequencies from parents of DNA pools

Considering that it is more convenient to detect allele frequencies of parents of the pools, the correlation between allele frequencies in the four different pools or individuals in the pools and their corresponding parents were analyzed (Table 3). The results showed that the correlation coefficients between allele frequencies of different pools or individuals in each pool and their corresponding parents were higher than 0.95, even in the smallest pool with 15 individuals. When the number of individuals in the pool reached 53, the correlation coefficients between allele frequencies of individuals in the pool or the mixed pool and their corresponding parents were more than 0.98. Compared with those of the pools, the allele frequencies of individuals in the pool were more correlated with those of their parents. With the increase of individuals and families, the correlation coefficient increased gradually. The effect of increasing the family number was more obvious than that of increasing the number of individuals (S3 Table).

**Table 3:** Correlation between allele frequencies of the four pools or of individuals in the pools and those of their corresponding parents.

Pool	Allele	4 pools and their corresponding parents		Individuals in the 4 pools and their corresponding parents	
		Pearson’s coefficient	Relative error	Pearson’s coefficient	Relative error
1	A	0.9500	0.2350±0.9439	0.9560	0.1623±0.4107
	T	0.9504	0.2345±0.9871	0.9566	0.1651±0.4094
	C	0.9496	0.1566±0.7712	0.9556	0.1146±0.3508
	G	0.9510	0.1526±0.7315	0.9561	0.1167±0.3606
2	A	0.9550	0.1738±0.5284	0.9600	0.1436±0.3756
	T	0.9563	0.1747±0.4961	0.9708	0.1441±0.3441
	C	0.9560	0.1263±0.5064	0.9600	0.1011±0.3106
	G	0.9566	0.1208±0.4113	0.9604	0.1016±0.3101
3	A	0.9804	0.3596±1.9266	0.9848	0.2310±0.8351
	T	0.9802	0.3558±1.3142	0.9847	0.2501±0.7928
	C	0.9800	0.2517±1.4403	0.9841	0.1638±0.6778
	G	0.9799	0.2442±1.0795	0.9844	0.1675±0.6624
4	A	0.9814	3.3075±55.0572	0.9874	1.6226±16.0181
	T	0.9812	3.1723±41.3763	0.9873	1.7100±18.6187
	C	0.9814	1.9864±25.1980	0.9871	0.8983±10.0046
	G	0.9816	1.8871±26.9650	0.9873	0.9837±12.0227

For the comparison of the 4 different pools and their corresponding parents, the relative error was 0.1526–0.2350 for pool 1, 0.1208–0.1747 for pool 2, 0.2442–0.3596 for pool 3 and 1.8871–3.3075 for pool 4. The relative error and its standard error increased as the number of individuals and families increased (Table 3). For the comparison of individuals in the 4 pools and their corresponding parents, the relative error was lower than that of the former, which was 0.1146–0.1651 for pool 1, 0.1011–0.1441 for pool 2, 0.1638–0.2501 for pool 3, and 0.8983–1.7100 for pool 4. When the family number was fixed (pool 1 and pool 2), the relative error was higher when the number of individuals increased. When the number of individuals was fixed (pool 2 and pool 3), the relative error was higher when the number of families increased (S4 Table).

### The effect of SNP number on the consistency of allele frequencies between different pools and their constituent individuals

**Fig 1:**
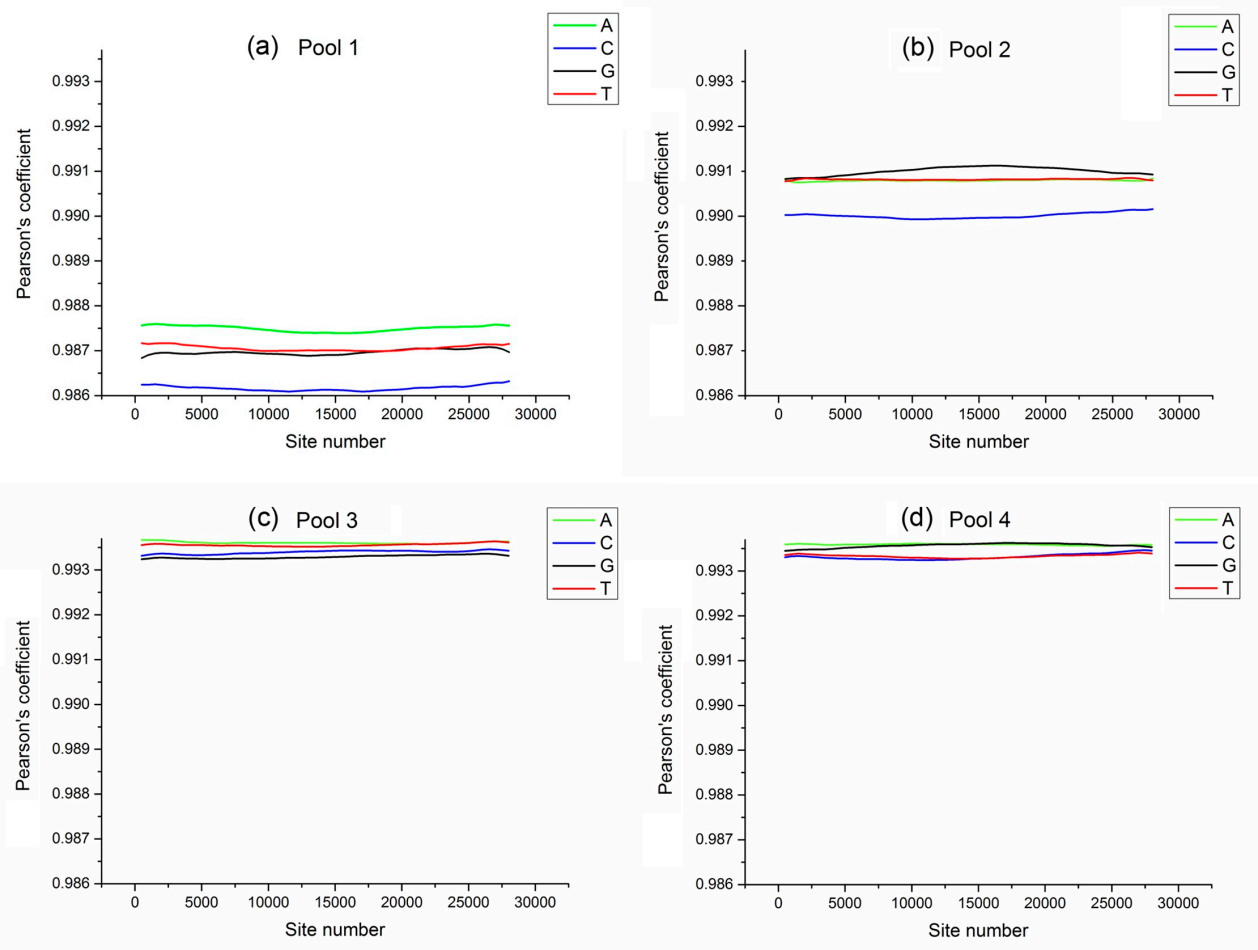
Correlation between site number and Pearson’s coefficients of allele frequencies between pool- and individual- sequencing. (a) Pool 1 that contains three families (F1-F3), each with five individuals. (b) Pool 2 that contains the same three families with pool 1, each with 10 individuals including those used in Pool 1. (c) Pool 3 that contains six families (F1-F6), each with five individuals, and eight of the fifteen individuals from F1-F3 were the same as those in Pool 1. (d) Pool 4 that contained ten families, including all 53 individuals used in Pool 1, Pool 2 and Pool 3. The x-axis and y-axis categorizations correspond to site number and Pearson’s coefficient of allele frequencies between pool- and individual- sequence, respectively.

In the four mixed pools, the correlation between SNP number and correlation coefficients of allele frequencies between pool- and individual- sequencing were relatively high (> 0.98), but there was no linear relationship between them. The detailed trends are shown in Fig 1. When the individual number in the pool increased, the standard deviation showed a downward trend. When the number of SNPs was fixed, the larger the number of individuals, the smaller the variation range of the pool sequencing allele frequency. The number of SNPs required for standard deviation < 0.0001 is shown in Table 4.

**Table 4:** SNP number required for standard deviation < 0.0001 of the correlation coefficients of allele frequencies between pool- and individual- sequence under a given SNP number.

	A	T	C	G
Pool 1	18,000	24,000	27,000	26,500
Pool 2	22,500	12,500	24,000	24,500
Pool 3	17,000	15,500	21,500	24,000
Pool 4	16,500	13,500	13,500	24,000

### Verification of pool sequencing repeatability

Correlations of allele frequency estimated between pool DNA and individuals for repeats of pool 3 and pool 5 were shown in Table 5. The results showed among three repeats, the standard deviations of Pearson correlation coefficient were less than 0.001 for both pool 3 and pool 5. The standard deviations of the mean relative errors were below 0.1. The allele frequency correlation of four bases was highly consistent between pool and individual sequencing, whether compared with all 53 individuals or the composition individuals of the pool (S5–S7 Tables).

**Table 5:** Correlations of allele frequency estimated between pool DNA and individuals for 3 repeats of pool 3 and pool 5.

Pool	Allele	2 pools and 53 individuals		2 pools and the constituent individuals	
		Pearson’s coefficient	Relative error*	Pearson’s coefficient	Relative error*
3	A	0.9929±0.0006	0.9187±0.0672	0.9944±0.0007	0.4676±0.0219
	T	0.9928±0.0006	0.8505±0.0108	0.9944±0.0007	0.4548±0.0066
	C	0.9929±0.0006	0.6127±0.0197	0.9943±0.0008	0.3136±0.0184
	G	0.9928±0.0008	0.5869±0.0487	0.9944±0.0009	0.3111±0.0303
5	A	0.9937±0.0004	0.7754±0.0385	0.9948±0.0004	0.4311±0.0222
	T	0.9937±0.0002	0.7669±0.0608	0.9948±0.0002	0.4282±0.0180
	C	0.9937±0.0003	0.6751±0.0195	0.9947±0.0003	0.3414±0.0187
	G	0.9937±0.0003	0.5646±0.0445	0.9949±0.0003	0.3292±0.0199

* The standard deviation was of the mean of three repeats.

Correlations between allele frequencies of pool 3, pool 5 or of individuals in the pools and those of their corresponding parents were shown in Table 6. The results showed among three repeats, the standard deviations of Pearson correlation coefficient were less than 0.01 for both pool 3 and pool 5. The standard deviations of the mean relative errors were below 0.1. Correlations between allele frequencies of individuals in the 2 pools and their corresponding parents were the same in three repeats (S5–S7 Tables). The allele frequency correlations of four bases were highly consistent between pool DNA and their parents. The standard deviations of Pearson correlation coefficient were less than 0.01 for both pool 3 and pool 5. The standard deviations of the mean relative errors were below 0.1. Correlations between allele frequencies of individuals in the 2 pools and their corresponding parents were the same in three repeats (S5–S7 Tables).

**Table 6:** Correlations between allele frequencies of pool 3, pool 5 or of individuals in the pools and those of their corresponding parents.

Pool	Allele	2 pools and their corresponding parents		Individuals in the 2 pools and their corresponding parents	
		Pearson’s coefficient	Relative error	Pearson’s coefficient	Relative error
3	A	0.9819±0.0013	0.3195±0.0353	0.9848	0.2310
	T	0.9820±0.0015	0.3216±0.0306	0.9847	0.2501
	C	0.9818±0.0015	0.2223±0.0263	0.9841	0.1638
	G	0.9819±0.0017	0.2237±0.0183	0.9844	0.1675
5	A	0.9838±0.0005	1.1506±0.0580	0.9864	0.7794
	T	0.9839±0.0002	1.2009±0.0412	0.9865	0.8438
	C	0.9839±0.0004	0.7826±0.0370	0.9864	0.4625
	G	0.9840±0.0004	0.7058±0.0256	0.9863	0.4505

## Discussion

Pooled DNA genotyping can be used to estimate the proportional contributions from multiple families in a pooled breeding population [21, 32], which is quite attractive to aquaculture selective breeding, where the value of a single individual is very low while family numbers are very large. Pool-seq was suggested to provide even more accurate allele frequency estimation than sequencing of individuals in previous studies [27, 33], because very few chromosomes are sequenced more than once in pool-seq, whereas for sequencing of individuals, each chromosome is typically sequenced multiple times (5–40 times) [34]. However, there are still many factors that need to be addressed in the application of pool-DNA sequencing, such as sequencing method, pool sizes, SNP numbers and so on. For example, if the restriction site contains polymorphisms in linkage disequilibrium with nearby SNPs, RAD-seq-based allele frequency may be biased because a polymorphic restriction site will only be cut in a fraction of the individuals [23, 35]. When RAD-seq analyses are carried out on pooled DNA samples, the problem may be more obvious because missing reads are much harder to spot and inevitably lead to biased allele frequency estimates. It is not clear to what extent this problem affects the accuracy of pooled DNA sequencing. In this study, 2b-RAD sequencing was used to assess the accuracy of allele frequency estimates obtained from 4 different pooled-DNA shrimp samples. Allele frequencies estimated from the pool were highly correlated with the ‘true’ allele frequencies obtained from the individual samples (*r* > 0.98), which showed 2b-RAD for pooled-DNA sequencing can obtain high-accuracy results.

The number of individuals and families in the pool has a direct impact on the accuracy of allele frequency estimation. Zhu et al. [24] found that when a sufficient number of strains were used in the pooling, the variation in the amount of DNA derived from individual strains as a substantial source of noise would be decreased in *D*. *melanogaster* [24]. Schlötterer et al. [34] pointed out that small pool sizes (<50 individuals) would yield suboptimal results for allele frequency estimates of individual SNPs. The results of this study also supported this argument. With increased individual and family numbers in the pool, the accuracy of allele frequency estimation increased. When the number of individuals or families was small, the accuracy was reduced, but still quite high. In this study, a mixed pool of 15 individuals also reached high concordance (*r* > 0.99) between allele frequency estimates derived from individual genotyping and DNA pools. Ozerov et al. [26] also obtained similar results in Atlantic salmon with 35 mixed individuals. Pooled DNA sequencing could achieve high accuracy, and the accuracy would increase with the number of individuals and families.

A total of 28,882 SNPs were used in this study. We found that using different numbers of SNPs (varied from 500 to 28,800) did not have a significant impact on the accuracy of pooled-DNA sequence. No positive correlation was found between the SNP number and accuracy of pooled-DNA seq. That is, fewer SNPs can also achieve good results but optimally chosen markers may increase performance. For example, low to 63 loci can achieve correlations between allele frequency estimates from pooled DNA and individual samples greater than 0.90 [32], and a correlation as high as 99% between estimated and true genetic contributions was achieved using 20 randomly chosen SNPs by simulation [21]. More markers (e.g., thousands) were thought to be needed for more families in the group and could potentially give highly accurate results in general.

Many studies have been done on other aspects that may affect the accuracy of mixed-pool sequencing, such as various sampling, sequencing depth, and experimental error designs and different library preparation protocols. Almost all theoretical and real data analysis ensured that pooled genotyping provided a cost-effective approach for estimating allele frequencies. E.g., the consensus is that the impact of differential representation of individuals on the accuracy of allele frequency estimates is not large unless sample sizes are very small [23, 24, 27]. Pool coverage was an important factor in allele frequency estimation. The results of a mathematical derivation showed that in pooled sequencing of 20 individuals, the accuracy of allele frequency estimation was higher when the pool coverage reached 82 × (unequal contribution) than 10 × coverage per individual [23]. In this study, individual coverage reached 15 × and pool coverage of 15–53 individuals reached 100 ×. Referring to the results of previous studies, the sequencing depth used in this study was appropriate [23, 24].

So far, most pooled-DNA sequencing studies were done by quantifying and mixing individual DNA because it could make the individuals in the pool mixed more evenly. For a practical breeding population, mixing tissue samples directly would be more cost effective. However, the allele frequency estimation bias caused by uneven mixing of samples should not be ignored. This source of bias was thought to be largely corrected to some extent by increasing pool size or pool coverage [28, 36]. Similar conclusions have been confirmed in the research of Gautier et al. [33] by mathematical deviation that if one pooling is not mixed evenly, more individuals would be required to reach the same accuracy as in the evenly mixed pools.

At the end of the study, the reproducibility of pooled DNA genotyping by 2b-RAD was tested. Three repetitions were carried out on two pools composed of 30 individuals (pool 3 and pool 5). The results of different repetitions were very consistent, which showed the reliability of pooled DNA genotyping by 2b-RAD was very high at least when the individual number in the pool was relatively small. When more individuals pooled, for example, more than 50, further research is still needed.

In conclusion, pooled DNA genotyping by 2b-RAD sequencing achieved high accuracy in the Pacific white shrimp, and the accuracy increased with the number of individuals and families in the pool. The allele frequencies of the parents from each pool were highly correlated with those of the pools or the corresponding individuals in the pool. The SNP number (500–28,800 SNPs) in this study had no significant effect on the estimation of allele frequency in pooled DNA. The data showed that pooled-DNA genotyping could be promising for evaluating shrimp breeding in a cost-effective way.

## Supporting information

S1 Table Comparison between 4 pools and 53 individuals. (XLSX)

S2 Table Comparison between 4 pools and constituent individuals. (XLSX)

S3 Table Comparison between 4 pools and corresponding parents. (XLSX)

S4 Table Information in 4 pools and corresponding parents. (XLSX)

S5 Table Information of two repetitions of pool 3. (XLSX)

S6 Table Information of three repeats of pool 5 and individuals. (XLSX)

S7 Table Information of three repeats of pool 5 and the parents. (XLSX)
